# Role of Fluoride in Dentistry: A Narrative Review

**DOI:** 10.7759/cureus.50884

**Published:** 2023-12-21

**Authors:** Nikhil Mankar, Saloni Kumbhare, Pradnya Nikhade, Joyeeta Mahapatra, Paridhi Agrawal

**Affiliations:** 1 Conservative Dentistry and Endodontics, Sharad Pawar Dental College and Hospital, Datta Meghe Institute of Higher Education and Research, Wardha, IND

**Keywords:** narrative review, fluoride, composite resin, caries, fluorosis

## Abstract

Fluoride has performed a central role in the growth over the last fifty years. This report examines the present state of knowledge about fluoride's involvement in preventing dental caries. In recent years, our knowledge of the development of dental caries and the manner of operation of fluoride has been shifted. Dental caries is a constant procedure for enamel demineralization and remineralization, and fluoride plays an important part in this action by acting at the plaque-enamel contact. Fluoride's major method of action is now recognized as posteruptive. Fluoride's post-eruptive activity has led to the development of novel fluoride delivery systems. The importance of various fluoride delivery techniques on a population and societal level is discussed, along with suggestions.

## Introduction and background

In dentistry, fluoride particles are crucial in protecting teeth to prevent decay. It is reported to contribute to the conventional tooth mineral's particular mineralization cycle and connect utilizing the mineralization, making a thin layer coating of fluorapatite. It's more impervious to pitting corrosion than adjacent hydroxyapatite, thus safeguarding the teeth from increased deterioration. One of the most popular dental restoratives is glass-ionomer dental concrete, which may supply fluoride consistently and can last for a prolonged period, which is considered therapeutically beneficial. Similarly, the gum-adjusted glass-ionomer compound and the equipment composite gum ("compomer") can be used [1]. There are far too many conventional composite materials containing fluoride that are fine to provide fluoride. These Inorganic materials are researched, as well as the method in which they distribute fluoride and the sufficiency of the delivery at the amounts in concern. The durability of fluoride release from these various types of materials is investigated and presented, implying that delivery from basic and tar-adjusted glass-ionomers is much more advantageous than delivery from composite gums for two main reasons: first, which is unable to replace something that has been lost in fluoride in composites, identical glass- ionomers, second, additional particles free of glass- ionomers might lead adjacent remineralization of the tooth. Because of the shortage of these distinct particles in fluoride composites, remineralization is less likely to occur [2].

## Review

Methodology

Both Medline via PubMed and the Central Database via the Cochrane Library were searched. Our keyword list included terms like "fluoride," "dentistry," "fluorosis," "effects," "oral," and "dental." We also looked for any new review articles in the reference lists of potentially pertinent papers. Our review included review articles that were located through these computerized searches as well as pertinent citations from those studies' bibliographies.

Fluoride consumption sources

Fluoride has mostly been obtained via food before the 1960s. Fluoridation and fluoride-containing oral care products were introduced in the 1950s and 1970s, which improved the situation. In Developed Market Economies, fluoride can be found in aquatic, salt, and meals containing fluoride, drinks, infant cereals, and cereals formulae, fluoride supplementation, mouthwashes, toothpaste, and local fluorides. Furthermore, fluoride in water causes a ripple effect, in which beverages and food produced in fluoridated zones are accessible to the entire populace, even those living in non-fluoridated areas. When these different fluoride methodologies were joined, it created an incoherent fluoride distribution strategy. More coordinated and effective fluoride delivery systems could result in significant savings, optimum caries reduction, and reduced dental fluorosis, it is now clear [1]. Other fluoride media, such as salt, milk, tablets, toothpaste, gels, and varnishes, were developed, evaluated, and promoted as a result of massive breakthroughs in understanding how fluoride affects the caries process [2].

Mechanism of fluoride

**Figure 1:**
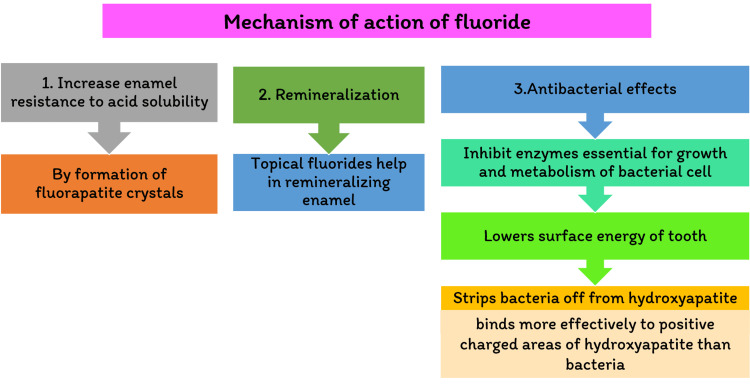
Flowchart showing the mechanism of action of fluoride

It was assumed that fluoride's mechanism was pre-eruptive (systemic) when it was initially introduced and for many years afterward. It was integrated into the affected tooth formation, resulting in a less fractured tooth soluble enamel apatite dissolving enamel. However, research on the mechanisms of a participant in tooth caries and the effect of fluoride has increased significantly. This notion has shifted our perception of it [1]. It is now widely accepted that fluoride is the main mechanism of action in preventing dental cavities. It is post-eruptive (topical), stimulating remineralization while inhibiting calcification. The caries process demineralizes tooth enamel, as shown in Figure 1.

Fluoride has several other effects, including preventing glycolysis (by acidifying the cytoplasm of cells and inhibiting enolase enzyme) and preventing tooth decay. Extracellular polysaccharide synthesis is decreased. Fluoride seems to have the power to possibly cariogenicity of Streptococcus mutans, and in large doses, it is antibacterial. The consequences of our modern understanding of fluoride's post-eruptive properties impact are that regular low-concentration fluoride exposure in the oral cavity is the most significant aspect of its usage in managing and preventing dental caries [2].

Role of fluoride with saliva

Post-eruptive [topical] fluoride delivery devices have been made due to this new understanding of fluoride's mode of action. The usefulness of possible antimicrobial protective actions (in non-experimental conditions) is less known. This lack of clarity may be attributable, at the very least in part, to ambiguity regarding the salivary sample that is an appropriate approach, in addition to the well-known methodological issues associated with connecting single salivary parameter measurements to the emergence of dental cavities in patients over two eras. The latter issue is solved by in vivo demineralization and remineralization systems in humans; however, it does not appear that the influence of individual salivary components on enamel demineralization and remineralization has been studied yet. The relevance of saliva in stabilizing plaque pH is clearly illustrated by Englander et al. (1959), who found that when saliva was removed from the plaque, the pH drop was higher and lasted longer than when saliva was permitted unrestricted access. Contrary to popular belief, calcium fluoride appears to be a critical step in understanding key features of something like the action of topically administered fluoride [3].

Salivary fluoride

Saliva's role in fluoride activity is now widely acknowledged. Fluoride can access the salivary system directly through meals or topical or passive therapies through the circulation either through the salivary glands or gingival crevicular fluid or just from transient intra-oral fluoride reservoirs such as calcium-fluoride-like surface deposits on the teeth. The persistent increase in salivary fluoride from average levels around 1µmol/L to roughly 2-5 µmol/L is usually regarded as the key therapeutic ingredient in preventing dental caries. It is probable that salivary and plaque fluoride equilibria exist that play a role in fluoride's cariostatic regulation. Clinical investigations and in situ model data show that the presence or absence of plaque does not influence fluoride remineralization (topical applications and rinses) (Dodds and Edgar 1991). Fluoride accumulation in plaque may be more essential in decreasing mineral depletion as opposed to increasing mineral acquisition since plaque is necessary for demineralization [4]. Minimal fluoride doses known to impact caries are inadequate to have a meaningful impact through the older method [5].

Hydroxyapatite and fluorapatite

**Figure 2:**
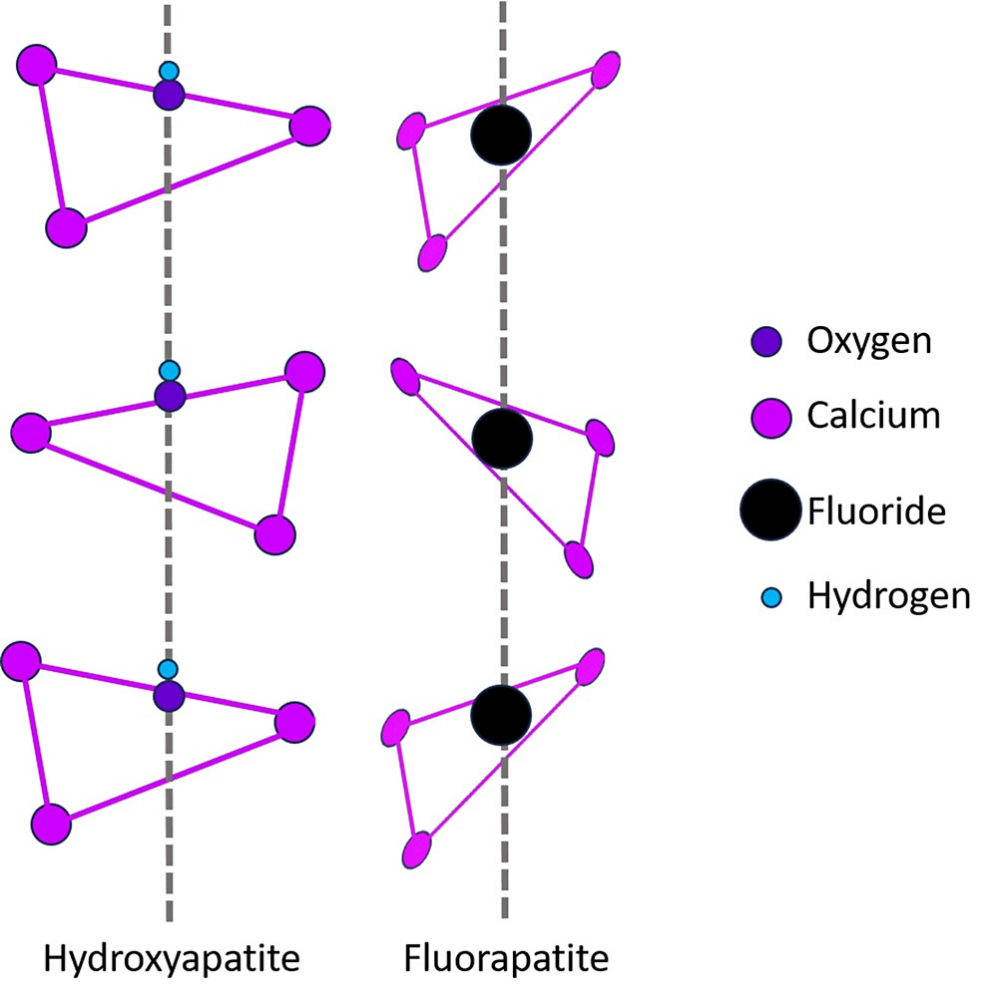
Structure of hydroxyapatite and fluorapatite

The calcium apatites hydroxyapatite (HA) and fluorapatite (FA) have the formula (Ca_10_(PO_4_)_6_X_2_) and are part of a group of calcium apatites. When hydroxyl groups are active, the mineral is called HA (Ca_10_(PO_4_)_6_(OH)_2_), and when fluoride ions are present, the mineral is called FA (Ca_10_(PO_4_)_6_F_2_), as shown in Figure 2.

The mineral hydroxyfluorapatite comprises both fluoride and hydroxyl ions (HFA). This kind of mineral's composition is then mentioned using the formula Ca_10_(PO_4_)_6_(OHxFy). The stoichiometric HA has the monoclinic shape P21/b, but multiple substitutions with other ions result in the hexagonal form P63/m in nature. The hexagonal structure of FA is P63/m [6]. Unit cells a and c have similar mineral characteristics, with a = 9.43 and c = 6.88 for HA and a = 9.37 and c = 6.87 for FA. FA has slightly smaller parameters a and c than HA, owing to the larger radius of the hydroxyl ion in FA. The mineral is known as HA (Ca_10_(PO_4_)_6_(OH)_2_), where hydroxyl groups are present, and FA (Ca_10_(PO_4_)_6_F_2_) once fluoride is present in high concentrations [7].

Role of fluoride in the maintenance of oral hygiene

Fluoride levels in the mouth are linked to the frequency and incidence of dental caries. The fluoride ion can prevent caries through various methods, including impacts on bacterial metabolism, albeit these are unlikely to be the predominant factors. It also affects the production of extracellular polysaccharides, the sugar transport system, enolase, and Adenosine triphosphatase (ATPase); however, unlike Sodium Fluoride (NaF), it has less impact on these systems. The fluoride ion's main action is to promote remineralization of early caries and to prevent demineralization; even minute levels have a big impact on both processes. On the other hand, sodium monofluorophosphate (MFP) must be broken down in the mouth to release fluoride, whereas the fluoride ion in sodium fluoride is immediately and completely accessible. MFP will release fluoride extremely slowly in the absence of phosphatases in an in vitro setting and will not have the same impact as NaF. Increased numbers of cariogenic bacteria such as Streptococcus mutans and Lactobacilli are detected in patients who ingest too much sugar.

Even a minor change in terminal pH and acid generation rate can stabilize plaque flora and remove some of the benefits of cariogenic (aciduric) species. Low fluoride ion concentration enhances remineralization of enamel defects in vitro. The amounts detected in saliva (0.01-0.03 ppm) are about in the range when solubility inhibition and remineralization enhancement begin to occur. Fluoride concentrations in plaque may hinder bacterial growth. Fluoride concentrations in plaque may impede acid generation by plaque organisms and elicit other metabolic effects. Plaque fluoride is a major reservoir in the mouth that stores fluoride for a long time. The increased total fluoride content in plaque compared to saliva is due to calcium fluoride's subsequent retention in the plaque. After using sodium fluoride dentifrices at varied doses, plaque and saliva fluoride levels are consistent with the established relationship between fluoride levels and clinical impact. Phosphatases, on the other hand, hydrolyze MFP relatively slowly on the tooth surface in vivo. As a result, fluoride levels in plaque are lower than in people who take Na [8]. We have to concentrate on 2 very closely related factors: (1) brushing teeth regularly and (2) post-brushing practice [9].

Role of fluoride in dental restorative materials

**Figure 3:**
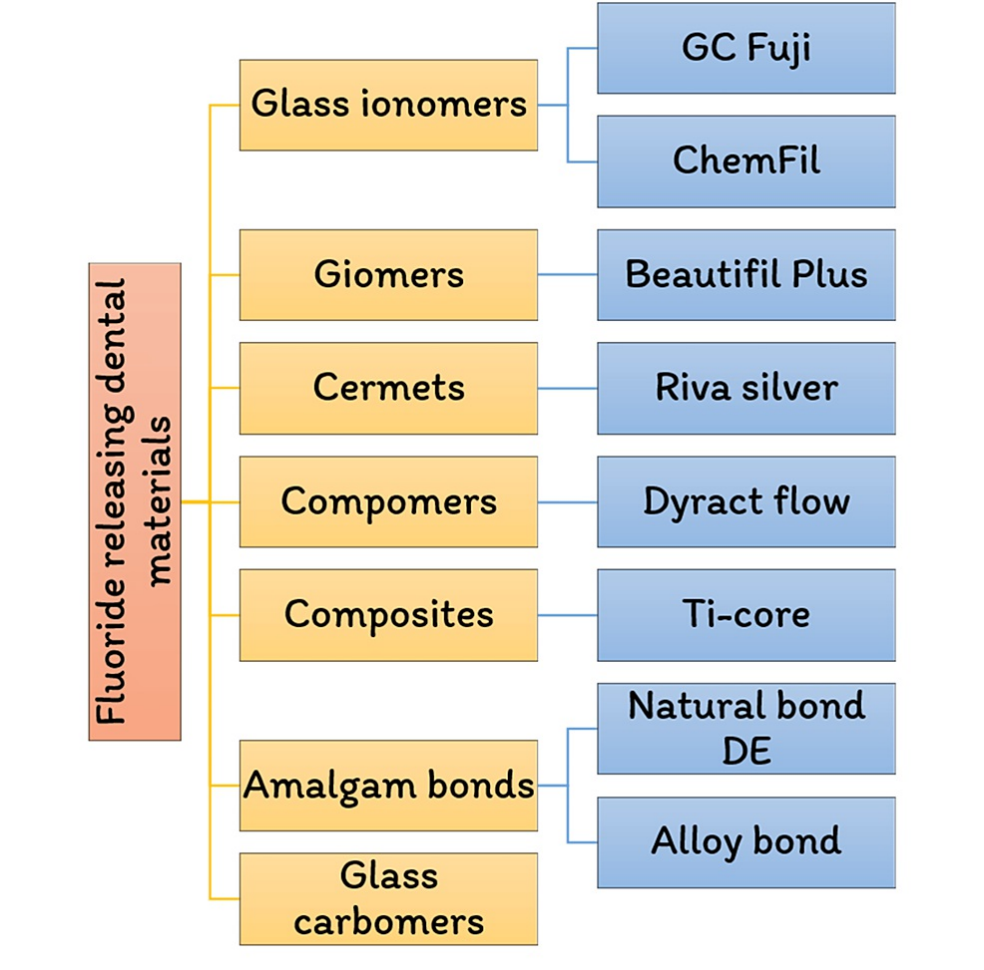
List of fluoride-containing dental materials

Microbes may erode tooth enamel, the strongest substance in the body. Gum disease is becoming most common in all populations worldwide [10]. Since the beginning of discovery in the 1930s, connected fluorosis has been a condition that occurs when there is a small amount of tooth deterioration. Fluoride has been recognized to play a function in preventing caries in the mouth. This problem has been extensively researched throughout the years, and it is now recognized that fluoride works topically rather than systemically. Long-term fluoride to which the teeth are exposed has also been proven to be the most efficient strategy to leverage this topical impact and reduce dental caries. The modern restorative materials are divided into various categories, as shown in Figure 3.

Composite Resins

They comprise complex organic monomers, such as bis- phenolglycidyl methacrylate (bis-GMA), and less viscous monomers, like triethylene glycol di-methacrylate. Direct composite bonding maintains an adequate therapeutic method in classic Class III to Class V, including localized aesthetic restorations [11]. Most novel composite materials have emerged as one-component paste applications that polymerize via photo initiation. Even though the finished product is appealing, it does not stick to the tooth's surface. Rather, it necessitates the use of a specific bonding agent. Composite resins are not fluoride-liberated by nature, although fluoride chemicals can be added to make them fluoride-releasing.

Polyacid-modified Composite Resins

These resources are created in the hopes of creating a composite resin that could release fluoride like traditional glass-ionomer cement. Diluents, large monomer molecules, and particle inorganic fillers are all present in them, just as they are in typical composite resins. They also include acid-functional monomers and a tiny quantity of reactive alumina silicate glass as a filler. After that, they'll be able to suck little large amount of saliva in the mouth, which activates the acid-base interaction, allowing the fluoride previously contained in the glass filler to be transported to the restoration's exterior. When these materials are exposed to moisture, they lose some of their mechanical properties, showing that the acid-base interaction does not affect strength. Because of their fluoride-releasing properties, these restoratives are commonly used in pediatric dentistry. Polyacid-modified natural polymers (compomers) are endodontically treated teeth products that share some characteristics with regular composite resins and glass-ionomer cement [12].

Glass Ionomer Cement

A calcium (or strontium) fluoroalumino-silicate, a glass grind, is combined with the catalyst polyalkenoic acid, commonly poly (acrylic acid), to create cement. This material hardens quickly, forming a porcelain-like substance that sticks to the tooth. Fluoride, as well as other ions (calcium or strontium, sodium, silicate, and phosphate), can be released by this structure. The ability of such materials to discharge ions is significant since this process is key in creating long-term permanent bonds with the tooth. Ions can migrate through into the interfacial area from the cement and the tooth, generating an ion exchange layer that seems to be exceptionally durable in clinical use. The spectrum of circumstances addressed using the ART technique demonstrates that the mechanical characteristics of set cement are adequate for various clinical applications. Despite these accomplishments, many authorities are wary of the widespread use of glass ionomers in restorative dentistry, and these materials are most often employed in pediatric dentistry, where their capacity to release fluoride is seen as a distinct benefit.

Resin-modified Glass Ionomers

Resin-modified glass ionomers were comparable to glass ionomers in that they comprise a water-soluble polymeric acid. However, they include 2-hydroxyethyl methacrylate (HEMA), a water-soluble organic monomer. To introduce HEMA addition polymerization, an initiator process is vital. It might be used for other purposes in other ways for core buildup and luting, although it requires a 2-part initiator. When the constituents get combined, ions are released rather than being photocured. This implies that they can be set in the dark. They are commonly utilized as amalgam replacements, notably in pediatric dentistry. According to the composition in the matrix material of the formed material, most of them have structural qualities that fall in between composites and standard glass-ionomers [13].

Giomers and Glass Carbomers

The giomers and the glass carbomers are two more tooth-colored materials that are touted to be novel in current clinical dentistry. As previously said, they are all sorts of established content. Giomers are a form of composite resin that flows, with pre-reacted ionomer glass coated with poly(acrylic acid) as at least part of the filler. Their antibacterial properties have been linked to their fluoride release [14].

Over-the-counter fluoride-containing agents

Fluoride Toothpaste

There are high-quality indication resources to assist the use of fluoride toothpaste in preventing caries. Fluoride toothpastes such as Cheerio gel, Colgate sensitive, Close up deep action, Senquel-F, and Sensodent-KF have always been the subject of well-controlled clinical trials showing caries reductions of up to 30%. Fluoride content in toothpaste varies from 1,000 to 1,100 parts per million. High-concentration toothpastes with 1,500 ppm may have had a minor additive advantage, but they aren't used as frequently as they should be low-fluoride toothpastes, such as 250 ppm fluoride, have been found to be ineffective. Caries decreased with toothpaste containing 500 to 550ppm fluoride, but not as much as with regular concentration toothpaste. Fluorosis is reduced when these low-concentration toothpastes are used. When compared with the use of usual fluoride-containing (1350 ppm F) toothpaste, using just a high fluoride having dentifrice (5000 ppm F) twice provides a smoother stiffness of somewhat untreated secondary caries [15].

Fluoride Gels and Varnishes

To reduce caries in youngsters, a sufficient level of concentration gels that contains 12,300ppm fluoride are employed. The processes involve a four-minute application, with measures designed to minimize fluoride consumption. Fluoride gels are only for high-risk individuals and aren't meant for Programs aimed at society or the general citizenry's welfare. Fluoride varnish has become much more easily obtainable in recent years. Normally, a high concentration of fluoride sodium fluoride (22,000ppm) is utilized (5%). Varnishes such as 3M™ Clinpro™ XT Varnish, GC MI Fluoride Varnish, Duraflor, and MiVarnish have been demonstrated to work at these doses and are used to treat hypersensitive patients. Fluoride varnishes were advised to people with acute, apprehensive patients with aneurysms and the clinically and physiologically impaired for infant and toddler caries, root-caries, and patients with orthodontic devices since the varnish is easy to implement. Fluoride varnishes bind onto enamel; thus, the calcium fluoride that forms subsequent absorption works as a protracted fluoride repository [16].

Fluoride Mouthrinses

Fluoride mouth rinses had been tested to benefit both a group and an individual. There are two basic methods for administering rinses: low-potency sodium fluoride (0.05%) and high-potency sodium fluoride (0.2%) [17]. The former is delivered every day, as the latter is delivered every two weeks. According to the recommendations, fluoride mouth rinses should not be used in children under six or only in high-risk youngsters. They are also suitable for those who wear orthodontic appliances or receive high radiation doses. Because alternate sources of fluoride are now readily available, its use as a school-based system is debatable [18].

Fluoride Supplements

Fluoride additives have been utilized for both the well-being of the general society and individual consumption. The recommended dose schedule differs by area [19]. When it comes to fluoride supplements, one must weigh problems of efficacy and a clear fluorosis hazard against the teeth-proven efficacy of systems like water fluoridation, which also has a lesser hazard of fluorosis. Fluoride supplements are used in certain situations of non-fluoridated areas in children aged 3 and up with a high risk of caries [20].

Slow Release Fluorides

Various technologies have been developed to allow fluoride to be gradually supplied in the oral cavity. Examples are co-polymer membrane gadgets, such as Fluoride glass appliances, which may produce fluoride gradually for up to a year and release fluoride for 6 months. It has a large amount of capability for people with special needs, but now items aren't readily approachable [21].

Dental fluorosis

**Figure 4:**
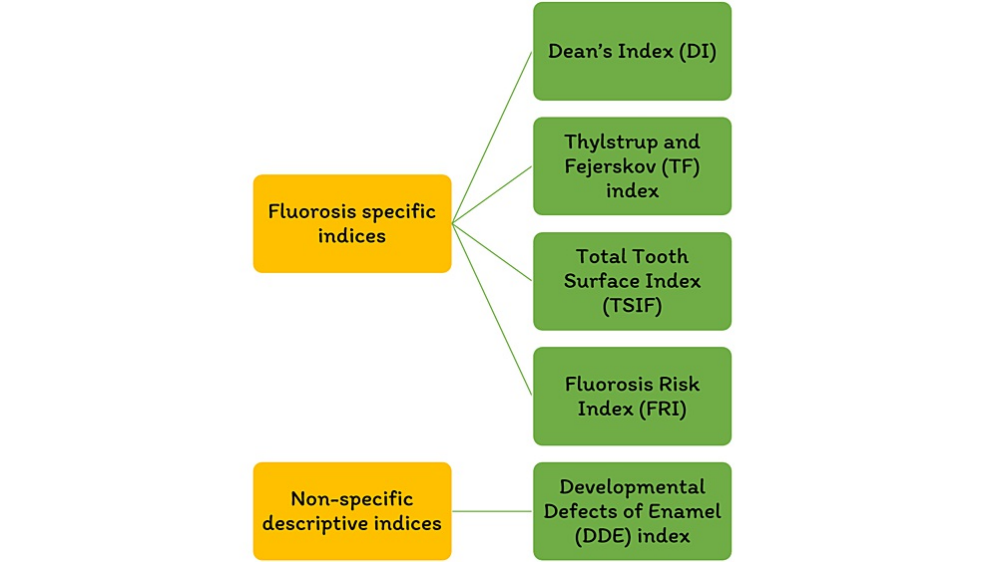
Various fluorosis index

**Figure 5:**
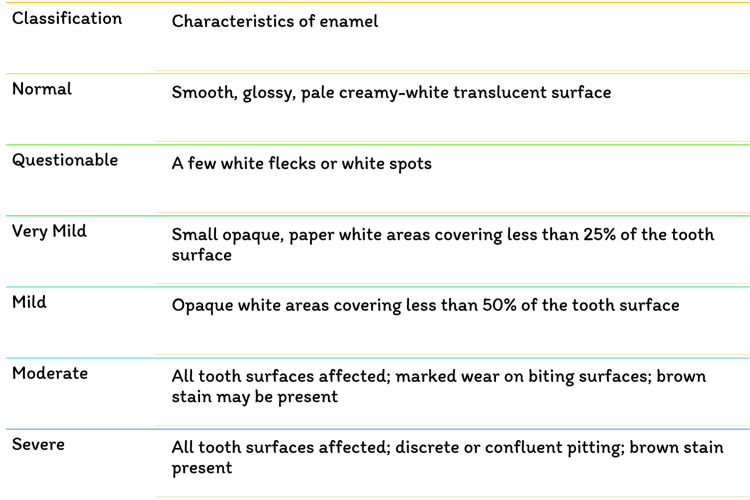
Dean's fluorosis index

**Figure 6:**
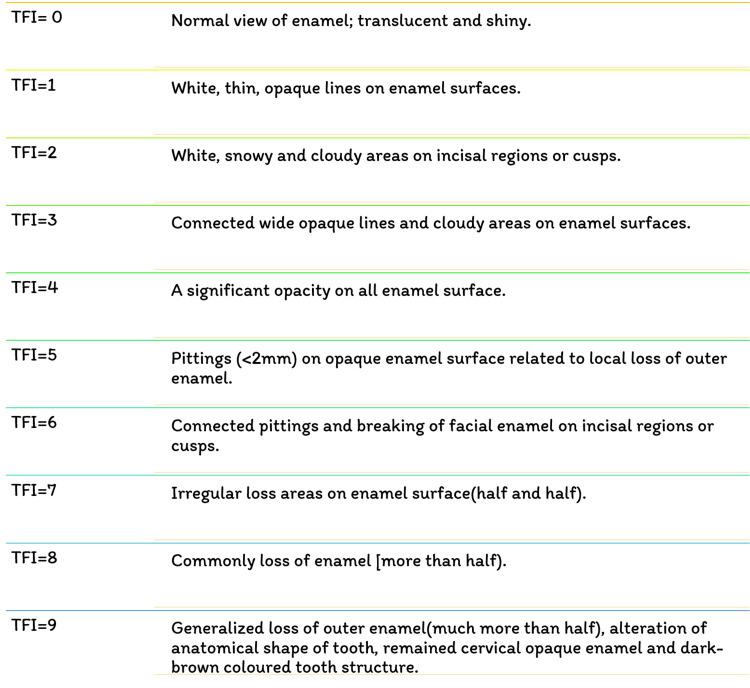
Thylstrup and Fejerskov Index TFI- Thylstrup and Fejerskov Index

**Figure 7:**
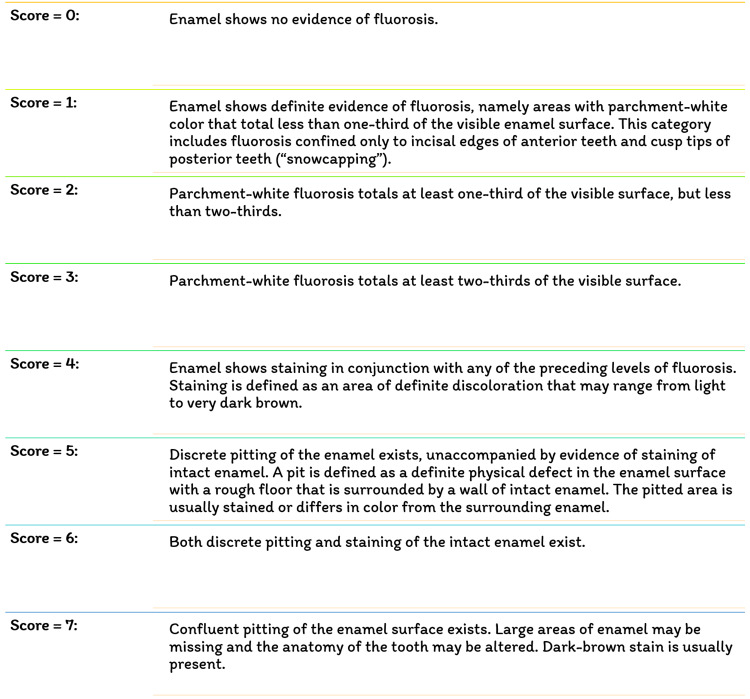
Total tooth surface index

Fluoride has been identified as the single most important component in the development of enamel mottling [22]. However, enamel mottling can be caused by inherent and/or extrinsic causes. Chronic acidosis and hypoxia generate enamel opacities in rats that seem histopathologically significant, identical to enamel fluorosis, regardless of the dose of fluoride exposure [23]. Milder types of enamel fluorosis are distinguished by narrow white lines that follow the perikymata, cuspal snow capping look that inadequacy a defined boundary by unaffected enamel. Along the surface of the enamel, there is a layer of underlying enamel in which the entire tooth becomes progressively. As the lesion advances, it spreads to the underlying enamel, causing the fluoride concentration to grow [24]. Dividing and larger surface enamel damage are now usually regarded as post-eruptive symptoms instead of true hypoplasia of the teeth; however, attrition and abrasion of hypo mineralized enamel, or possibly reduced mineral assimilation, may lessen the degree of the weaker hypoplasia [25]. Figure 4 depicts the various fluorosis indices. Figure 5 shows the dean's fluorosis index. Figure 6 shows Thylstrup and Fejerskov Index. Figure 7 shows total tooth surface index.

A moderately fluorosed tooth structure discovered that the nutritional quality of fluorosed enamel was higher than the regular enamel (mean fluorosed, 0.27 percent vs. 0.11 percent); the amino acid statuses are the same, so there was no indication of the utilization of immature matrix proteins in the test [26-28]. The evident discrepancy between the two studies could be linked to the fact that each report stored and processed the samples individually. There is still a scarcity of statistical methods on the proteins that remain in regular or fluorosis enamel [29]. Human physiological and metabolic variables, dietary composition, fluoride bioavailability, ambient fluoride, and environment could all play an impact. When consumed, fluoride is readily taken up by the blood plasma, primarily in the abdomen. Consequently, the amount and type of food consumed in the stomach will have a massive effect on fluoride absorption [30].

## Conclusions

The role of fluoride in dentistry is pivotal in protecting teeth and preventing dental decay. Over the years, extensive research has unveiled the complex mechanisms by which fluoride operates to maintain oral health. Fluoride's effectiveness is primarily post-eruptive, involving topical applications that stimulate remineralization and inhibit demineralization. It promotes enamel remineralization, reduces glycolysis, and exerts antibacterial effects, particularly against cariogenic bacteria like Streptococcus mutans. Fluoride's influence on saliva is also critical, as it maintains a low, constant concentration of fluoride in the oral cavity, aiding in preventing dental caries. Using fluoride-containing dental materials plays a crucial role in maintaining oral health. In addition to restorative materials, fluoride is delivered through dental products like toothpaste, gels, varnishes, and mouth rinses. These products have been shown to reduce the incidence of caries, particularly in high-risk individuals. Fluoride supplements are also available for individuals in non-fluoridated areas, further expanding the reach of fluoride's benefits. It is important to note that while fluoride offers significant advantages in dental health, there is a risk of dental fluorosis, which can result from excessive fluoride exposure during tooth development. Thus, proper fluoride monitoring and application are essential to balance caries prevention and the potential for cosmetic issues.
